# Unaltered myocilin expression in the blood of primary open angle glaucoma patients

**Published:** 2012-04-21

**Authors:** Khaled K. Abu-Amero, Taif Anwar Azad, George L. Spaeth, Jonathan Myers, L. Jay Katz, Marlene Moster, Thomas M. Bosley

**Affiliations:** 1 Department of Ophthalmology, College of Medicine, King Saud University, Riyadh, Saudi Arabia; 2 Department of Ophthalmology, College of Medicine, University of Florida, Jacksonville, FL; 3 William and Anna Goldberg Glaucoma Service, Wills Eye Hospital, Thomas Jefferson University, Philadelphia, PA

## Abstract

**Purpose:**

To investigate the expression of the myocilin gene (*MYOC*) in the blood of primary open angle glaucoma (POAG) patients to determine if altered systemic expression is playing a role.

**Methods:**

Patients (n=47) were eligible for inclusion if they met standard clinical criteria for POAG. Control subjects (n=27) were recruited who were free from glaucoma by examination. RNA was extracted from leukocytes of patients and controls and converted to cDNA by reverse transcriptase enzyme, and quantitative PCR was used to assess expression levels of *MYOC* and the house keeping gene β-globulin (*HBB*). The ratio of *MYOC* expression to *HBB* expression for POAG patients was compared to that of controls and to clinical characteristics of POAG patients.

**Results:**

Mean gene expression values were statistically similar in POAG patients and controls for both *MYOC* (p≤0.55) and *HBB* (p≤0.48). *MYOC*/*HBB* ratios were also statistically indistinguishable between POAG patients and controls (p≤0.90). *MYOC*/*HBB* ratios were not significantly associated with age, sex, or ethnicity of patients within the POAG group. Similarly, *MYOC*/*HBB* ratios were not significantly associated with clinical parameters related to POAG severity, including maximum intraocular pressure, vertical cup-to-disk ratio, static perimetry mean deviation, or static perimetry pattern standard deviation.

**Conclusions:**

*MYOC* expression is not altered in the blood of POAG patients, unlike *MYOC* expression in trabecular meshwork (TM) cultures. These results suggests that *MYOC* expression is not altered systemically but rather that *MYOC* expression may contribute to POAG pathogenesis in specific tissues such as TM.

## Introduction

Glaucoma is one of the leading causes of blindness worldwide [1,2], characterized by chronic degeneration of axons in the optic nerve head. Primary open angle glaucoma (POAG) is the most prevalent type of glaucoma in western countries and has risk factors that include elevated intraocular pressure (IOP) and age [3]. Elevated IOP is associated with increased aqueous humor outflow resistance in the trabecular meshwork (TM) of the eye [2], although the exact mechanism and causative factors for this increase is unclear. Up to half of all patients with POAG have a positive family history [4,5], and these and other observations suggest that genetic factors may contribute to POAG [1,6,7].

Myocilin *(MYOC)* was the first gene linked to POAG [8] and is the one most studied [9]. It is located in chromosome 1, contains three exons, and codes for a largely extracellular matrix protein. This protein has an NH_2_-terminal coiled region and a COOH-terminal olfactomedin domain [10], but its function is still not well understood. To date, mutations in *MYOC* seem most likely to have their pathogenic effect largely because of inability of the protein to fold properly [11]. This may result in an unfolded protein response in TM cells, activating a mitochondria-independent apoptosis pathway which ultimately leads to cell death, breakdown of TM cell structure, obstruction of aqueous humor outflow pathway, ocular hypertension, and ultimately the optic nerve damage of glaucoma [12-14]. *MYOC* may directly impair optic nerve function when mutated [15]; however, direct evidence for this hypothesis is still lacking [11,15].

Most studies investigating *MYOC* expression in POAG have employed human cultured TM cells [16,17]. However, whole blood gene expression studies have been used to investigate POAG [18], other hereditary optic neuropathies [19], and diseases affecting brain anatomy and function [20,21] because the target tissues for these diseases is not readily available. Therefore, the current study investigated *MYOC* expression in whole blood from POAG patients in hope that this approach will add to our knowledge of whether altered systemic expression of this gene contributes to POAG pathogenesis.

## Methods

### Patients and controls

Patients (n=47) were evaluated in the Glaucoma Service at the Wills Eye Institute, Philadelphia, PA, and enrolled after examination by a glaucoma specialist. Patients were eligible for inclusion if they met the following clinical criteria for POAG [22-25]: age greater than 40 years; intraocular pressure (IOP) ≥21 mmHg in one or both eyes before initiation of glaucoma treatment; normal-appearing, open anterior chamber angles bilaterally by gonioscopy; optic nerve appearance characteristic of the optic discs typically observed in primary open-angle glaucoma (with localized narrowing or absence of the neuro-retinal rim, with the amount of cupping exceeding the amount of pallor of the rim, and with asymmetric cupping of the optic discs in the two eyes); and static visual field (using a full threshold 24–2 program; Humphrey Field Analyzer II; Carl Zeiss Meditec, Inc., Dublin, CA) showing abnormalities typical of glaucoma (as per Advanced Glaucoma Intervention Study criteria) [26]. Good agreement was required between the appearance of the optic disc and the visual field. Exclusion criteria included historical, neuroimaging, or biochemical evidence of another possible optic neuropathic process affecting either eye, significant visual loss in both eyes not associated with glaucoma, or choosing not to participate. This research adhered to the tenets of the Declaration of Helsinki, and all patients and controls signed an informed consent approved by the Wills Eye Institute institutional review board.

All control subjects (n=27), frequently spouses of patients, had full ophthalmologic examinations documenting IOPs that were <21 mmHg and symmetric in the two eyes, normal anterior chambers, optic discs that were normal and symmetric in appearance, entirely normal static perimetry OU, and no prior history of glaucoma. All controls had static perimetry performed in the same fashion as POAG patients.

### DNA testing

Five ml of peripheral blood were collected in EDTA tubes from all participating individuals. DNA was extracted using the Illustra blood genomic Prep Mini Spin Kit from GE Healthcare (Buckinghamshire, UK), and stored at −20 °C in aliquots until required.

All patient and control DNA samples were tested for mutations in *MYOC* and its promoter. Successfully amplified fragments were sequenced in both directions using the M13 forward and reverse primers and the BigDye terminator v3.1 cycle sequencing kit (Applied Biosystems, Foster city, CA). Fragments were then run on the 3130*x*l Genetic Analyzer (Applied Biosystems) according to the manufacturer protocol. All sequenced fragments were then analyzed using SeqScape software v2.6 (Applied Biosystems). Table 1 details the sequence of the primers used the PCR annealing temperature and the expected amplicon size.

**Table 1:** Primer sequences, PCR annealing temperature and amplicon size for the myocilin gene.

**Exon**	**Primer sequence**	**Annealing temp (°C)**	**Amplicon size (bp)**
Pro-F	**TGTAAAACGACGGCCAGT**GCTGGCTGTTATTTTTCTCTGT	55	592
Pro-R	**CAGGAAACAGCTATGACC**CAGAAGCAGCAGCTGGACA		
1F	**TGTAAAACGACGGCCAGT**CACCCATCCAGGCACCTC	57	775
1R	**CAGGAAACAGCTATGACC**GAGCGCCTGTAGCAGGTCA		
2F	**TGTAAAACGACGGCCAGT**TGGCCGGCAGCCTATTTA	57	325
2R	**CAGGAAACAGCTATGACC**TGGGTGGGCATTTACCCTAT		
3AF	**TGTAAAACGACGGCCAGT**CATCTACTGGCTCTGCCAAG	57	850
3AR	**CAGGAAACAGCTATGACC**AAGTTGTCCCAGGCAAAGAG		
3BF	**TGTAAAACGACGGCCAGT**TGACTACAACCCCCTGGAGA	57	700
3BR	**CAGGAAACAGCTATGACC**CTGCAATCACATCTCCCAAC		

F=forward; R=reverse; Pro – promoter. Bold and underlined sequences are those of the M13.

### Quantitative RT–PCR

A two-step semi-quantitative reverse transcriptase  polymerase chain reaction (RT–PCR) method was used to measure gene expression levels of *MYOC* and β-globulin (*HBB*) in POAG patients and controls. Random hexameres were used as primers in the first step of cDNA synthesis. Total RNA (1 μg) was combined with 0.5 μg primers, 200 μM dNTPs, and sterile Milli-Q water (Millipore, Billerica, MA) and preheated at 65 °C for 2 min to denature secondary structures. The mixture was then cooled rapidly to 20 °C and then 10 μl 5× RT Buffer, 10 mM dithiothreitol, and 200 U Moloney Murine Leukemia Virus Reverse Transcriptase (Invitrogen Life Sciences, Grand Island, NY) were added for a total volume of 50 μl. The RT mix was incubated at 37 °C for 90 min then stopped by heating at 95 °C for 5 min. The cDNA stock was stored at −20 °C.

Relative RT–PCR was performed to measure gene expression of *MYOC* and *HBB* according to standard guidelines [27]. Primer sequences and optimal PCR annealing temperatures (ta) are listed in Table 2. Primer sequences were designed to span intron regions to insure that no false positive PCR fragments would be generated from pseudogenes and contaminate genomic DNA. In addition, all forward PCR primers were labeled with fluorescein (6-FAM), making quantitation more accurate. Polymerase chain reactions were performed using 100 ng of cDNA, 5 pmoles of each oligonucleotide primer, 200 μM of each dNTP, 1 unit of HotStar Taq-polymerase (Qiagen, Valencia, CA) and 1× PCR buffer in a 20 μl volume. The PCR program initially started with a 95 °C denaturation for 5 min, followed by 25 cycles of 95 °C for 1 min, ta °C for 45 s, and 72 °C for 1 min. Linear amplification range for each gene was tested on the adjusted cDNA, and 25 cycles were found to be optimal for both *MYOC* and *HBB*. The PCR samples were electrophoresed on the 3130*x*l Genetic Analyzer (Applied Biosystems).

**Table 2:** Primer sequences and annealing temperature β-globulin and myocilin fluorescent labeled primers.

**Primer name**	**Primer sequence**	**Annealing temp (°C)**
β-globulin-F	(6-FAM)AGCCTCGCCTTTGCCGA	57
β-globulin-R	CTGGTGCCTGGGGCG	
*MYOC*-LAB-F	(6-FAM)TTTCTACGTGGAATTTGGACA	59
*MYOC*-R	GTAGGTGGGCTTGGGGTCT	

F=forward; R=reverse. The forward primers were labeled with 6-FAM.

### Statistical analysis

Absolute RT–PCR values were used to calculate a ratio of the *MYOC* peak area in the selected linear amplification cycle divided by that of *HBB*, creating an *MYOC*/*HBB* ratio. All clinical and genetic data were analyzed using SPSS v17 (IBM, Chicago, IL).

## Results

Age (POAG patients 67.3 years; controls 63.6 years; p≤0.17) and sex (POAG 26 males/21 females; controls 12/15; p≤0.18) of the 47 unrelated POAG patients were similar to the 27 control individuals, but ethnicity differed between the POAG group (25 Caucasian/22 African American) and the control group (23 Caucasian/4 African American; p≤0.003).

After aligning and reading all sequences neither POAG patients nor controls were found to have any significant mutation or polymorphism in the coding or promoter regions of *MYOC*.

Mean gene expression values for both *MYOC* and *HBB* (p≤0.48) were statistically similar in POAG patients and controls (Table 3). *MYOC*/*HBB* ratios (p≤0.90) were also indistinguishable between POAG patients and controls. Because of ethnic differences between the POAG group and controls, gene expression values and ratios were also compared between Caucasian POAG patients and Caucasian controls. Mean *MYOC* (p≤0.90) gene expression and *MYOC*/*HBB* (p≤0.54) ratios also did not differ between these groups.

**Table 3:** Myocilin gene expression in POAG patients and controls.

**Parameter**	**Number POAG:control**	**POAG**	**Control**	**p≤**
*MYOC* expression; mean (SD)	47:26	7476 (4325)	6839 (4271)	0.55
β-globulin expression; mean (SD)	44:24	109533 (31355)	103885 (31047)	0.48
*MYOC*/β-globulin; mean (SD)	42:23	0.0685 (0.0520)	0.0667 (0.0597)	0.90
*MYOC* expression in Caucasians; mean (SD)	26:22	7132 (4526)	7268 (4121)	0.90
β-globulin expression in Caucasians; mean (SD)	22:20	104941 (31552)	99392 (32171)	0.58
*MYOC*/β-globulin in Caucasians; mean (SD)	21:19	0.0622 (0.0487)	0.0730 (0.0619)	0.54

POAG=primary open angle glaucoma; *MYOC*=myocilin gene expression; SD=standard deviation.

*MYOC*/*HBB* ratios were not significantly associated with age, sex, or ethnicity of patients within the POAG group (Table 4). Similarly, *MYOC*/*HBB* ratios were not significantly associated with clinical parameters related to POAG severity, including maximum intraocular pressure, vertical cup-to-disk ratio, static perimetry mean deviation, or static perimetry pattern standard deviation. Power calculations indicate a power ≤80% on these tests, leaving open the possibility of false negative type II statistical errors.

**Table 4:** Correlation between clinical parameters and MYOC/β-globulin ratios.

**Clinical parameter**	***MYOC*/β-globulin**	**p≤**
Age in years	0.204	0.21
Sex	0.040	0.81
Ethnicity	0.122	0.45
Visual acuity OD	0.016	0.92
Visual acuity OS	0.075	0.64
Maximum IOP OD	0.114	0.48
Maximum IOP OS	0.237	0.14
Vertical c/d ratio OD	0.129	0.43
Vertical c/d ratio OS	0.171	0.29
MD OD	−0.042	0.80
MD OS	−0.025	0.88
PSD OD	0.211	0.19
PSD OS	0.024	0.89

*MYOC*/β-globulin column contains correlation coefficients; OD=right eye; OS=left eye; IOP=intraocular pressure; c/d=cup to disk; MD=Humphrey visual field mean deviation; PSD=Humphrey visual field pattern standard deviation.

## Discussion

The 47 patients reported here met rigorous clinical criteria for POAG [22-25] with elevated IOP, normal anterior chamber, and evidence on funduscopic exam and visual fields of glaucomatous optic nerve damage. They did not have evidence of other types of glaucoma or alternative causes of optic nerve injury by clinical criteria, and none had dysmorphism or an obvious genetic syndrome. They were compared to 27 control individuals in whom POAG and other evidence of optic nerve damage were carefully excluded.

Screening the full *MYOC* gene and its promoter region revealed no mutations or significant polymorphisms in POAG patients or controls. These results are not surprising, since the prevalence of *MYOC* mutations is generally less than 5% in adult POAG populations [28]. Currently, there are 85 glaucoma causing mutations listed in the comprehensive myocilin database. They were classified as a glaucoma causing mutations based on the following criteria: i) predicted disruption of protein translation (e.g., frame-shift mutations and premature stop codons); ii) sequence variant frequency in control (unaffected) populations (those with a frequency >1% were classified as polymorphisms); iii) variant location (i.e., protein homology domain; cross species conservation of coding sequence); iv) evidence for partial segregation with the phenotype within a family and v) results of solubility studies. Interestingly, several sequence changes have been reported in the *MYOC* promoter region, but they were defined as neutral polymorphisms based on the pathologic characteristics described above (myocilin). This may indicate that the promoter region is free of mutations which could alter its expression. Alternatively, assessing potential pathogenicity for promoter region sequence changes can be challenging and may not follow the same pathological criteria as sequence changes found in coding regions [29].

Expression of *MYOC* in blood of POAG patients was unchanged compared to that of controls (Table 3). Expression was statistically similar to that of the housekeeping gene *HBB,* and the normalized expression of *MYOC* (*MYOC/HBB*) also did not differ between patients and controls. *MYOC* expression did not differ between Caucasian and African American POAG patients and ethnicity matched controls. Finally, *MYOC/HBB* did not correlate with age or with various clinical factors associated with POAG such as visual acuity, IOP, and C/D ratio (Table 4).

The lack of significant change in *MYOC* expression in blood of POAG patients stands in contrast to previous studies documenting decreased expression of *MYOC* in cultured human TM cells [16]. Gene expression studies in whole blood are clearly capable of documenting changes pertinent to complex neurologic diseases such as autism [30], amytrophic lateral sclerosis [31], schizophrenia [32],and other psychoses [33]. Patients with Leber hereditary optic neuropathy, another spontaneous optic neuropathy with a genetic pathophysiology, had decreased *OPA1* expression in blood [19]. Similarly, this same POAG patient and control group exhibited decreased systemic *OPA1* expression in blood [18]. *MYOC* expression is greatest in the TM [34] and is dramatically induced by dexamethasone, a glucocorticoid [17], in human TM cultures, leading to the original name for the *MYOC* protein of TM inducible glucocorticoid response element (TIGR) [35] and supporting the concept that *MYOC* may be responsible in part for steroid-induced glaucoma [36].

A potential limitation of this study is that the number of individuals studied was relatively small, bringing up the possibility that the lack of a statistical difference in *MYOC* expression and *MYOC/HBB* ratio between POAG patients and controls might be due to a type II statistical error because of inadequate power. This same patient group was adequate to confirm statistically significant differences in optic atrophy type 1 (*OPA1*) expression and the *OPA1*/*HBB* ratio between POAG patients and controls [18], but it is possible that differences in *MYOC* expression between POAG patients and controls are smaller, although still present. Similarly, the lack of correlation between the *MYOC*/*HBB* ratio and various clinical parameters within the POAG group may be subject to type II statistical errors. The population studied was predominantly Caucasian and African-American, and different results might be obtained in other ethnicities.

We found that systemic *MYOC* expression was unchanged in these POAG patients compared to controls. One interpretation of these results is that the *MYOC* protein plays a particularly important role in the globe and that regulation of *MYOC* expression that might be pertinent to POAG, congenital glaucoma, and/or steroid-induced glaucoma is relatively specific to the TM and may not be reflected to a significant extent in bone marrow or other non-ocular tissues. It is also possible that POAG is not altered by wild-type *MYOC* expression in any tissue [37]. A gain-of-function disease model was suggested after identification of mutant, misfolded forms of the *MYOC* protein were found aggregated in the endoplasmic reticulum of TM cells [38]. TM cells are essential for homeostatic regulation of aqueous humor, and their disruption may cause elevated intraocular pressure. A mutation-dependent, gain-of-function association between human *MYOC* and the peroxisomal targeting signal type 1 receptor (PTS1R) led to the [39] hypothesis that specific *MYOC* mutations may cause different amounts of MYOC misfolding, with corresponding varying degrees of recognition by the ubiquitin degradation pathway. A greater opportunity for mutant *MYOC* to interact with PTS1R may allow for poorer clearance from the TM endoplasmic reticulum and greater trabecular cell dysfunction, culminating in a higher IOP phenotype [39].
